# An approximate analytical solution of the Bethe equation for charged particles in the radiotherapeutic energy range

**DOI:** 10.1038/s41598-017-10554-0

**Published:** 2017-08-29

**Authors:** David Robert Grimes, Daniel R. Warren, Mike Partridge

**Affiliations:** Cancer Research UK/MRC Oxford Institute for Radiation Oncology, Gray Laboratory, University of Oxford, Old Road Campus Research Building, Off Roosevelt Drive, Oxford, OX3 7DQ UK

## Abstract

Charged particles such as protons and carbon ions are an increasingly important tool in radiotherapy. There are however unresolved physics issues impeding optimal implementation, including estimation of dose deposition in non-homogeneous tissue, an essential aspect of treatment optimization. Monte Carlo (MC) methods can be employed to estimate radiation profile, and whilst powerful, these are computationally expensive, limiting practicality. In this work, we start from fundamental physics in the form of the Bethe equation to yield a novel approximate analytical solution for particle range, energy and linear energy transfer (LET). The solution is given in terms of the exponential integral function with relativistic co-ordinate transform, allowing application at radiotherapeutic energy levels (50–350 MeV protons, 100–600 Mev/a.m.u carbon ions). Model results agreed closely for protons and carbon-ions (mean error within ≈1%) of literature values. Agreement was high along particle track, with some discrepancy manifesting at track-end. The model presented has applications within a charged particle radiotherapy optimization framework as a rapid method for dose and LET estimation, capable of accounting for heterogeneity in electron density and ionization potential.

## Introduction

The interaction of charged particles has long fascinated physicists; as early as 1903, William H Bragg demonstrated that a charged particle experiences maximal energy loss per unit path length just before it comes to rest^1^. The resulting relation for energy deposition as a function of depth, eponymously termed the Bragg curve, stands in stark contrast to the equivalent relation for photons, which exhibit quasi-exponential decay in energy transfer with depth. In the context of medical physics, the unique behaviour of charged particles offers the alluring possibility of radiotherapy dose deposition primarily at the location of a tumour within the body whilst sparing downstream tissue. There has consequently been significant clinical interest in modalities such as proton therapy^2–6^. In recent years, there has been interest in exploiting heavier charged particles such as carbon ions^7^ for clinical benefit, although there are significant engineering and cost challenges to overcome before this is widely implemented.

While proton therapy has distinct advantages relative to conventional photon treatment, uptake is still relatively limited. Despite many decades of interest, there is still a paucity of reliable clinical evidence and a need for more trials^8, 9^. In a recent review on the physics of proton therapy, Newhauser and Zhang^4^ enumerated a number of unresolved physics issues that need to be urgently addressed if this is to be more widely taken up - these include improved quantification of range and uncertainty, optimal strategies for treating tumours, dose estimation and the introduction of novel treatment paradigms such as proton arc therapy. Uncertainty analysis in planning also remains a pressing and distinct problem - tackling dose distribution in non-homogeneous human tissue has received little attention to date, despite this being a much greater concern in charged particle therapy relative to conventional X-ray treatment^10^.

Carbon ions have much greater economic and practical barriers to implementation, and currently only 5 facilities world-wide provide carbon treatment. While there is some clinical evidence of improved efficacy relative to x-rays and protons^11^, there is still a scarcity of data and scepticism over the economic and practical aspects of carbon therapy abounds^12^. Like proton radiotherapy, carbon therapy also needs significant research effort for similar reasons, in particular the estimation of dose through heterogeneous human tissue.

Quite aside from the technical challenges, there are also a number of theoretical physical and modeling problems that demand the attention of physicists and modellers. One of the most significant difficulties is adequately modeling tissue-particle interaction, and the computational power required for this. Modeling of radiation interaction is crucial for many applications, and if charged particles are to be exploited to their full potential it is imperative that energy deposition through different media is well understood. Monte Carlo (MC) techniques are typically employed for this purpose^13^. MC particle transport packages such as GEANT4^14^, MCNPX^15^ and FLUKA^16^ are extremely powerful and capable of simulating the interaction of particle radiation with matter as it traverses a medium. These approaches can capture system behaviour at all scales, including secondary and higher order interactions, but are computationally expensive with non-trivial evaluation times. Fast, approximate methods for estimating the energy profile through different media are especially useful in radiotherapy treatment planning, where multiple rapid dose calculations are needed to enable interactive optimization. More than this, such methods should ideally work with various particle energies and types, and be applicable not only to uniform media but also to the heterogenous nature of human tissue.

To achieve this, the purpose of this work is to derive an analytical model which captures the general behaviour of charged particles at the energy ranges pertinent to therapy (50–350 MeV for protons, 100–600 MeV/a.m.u for carbon ions). Previous authors have deduced useful functions to describe the Bragg curve in radiotherapy, based on empirically-determined range-energy relationships^17, 18^ which work well for low energy protons, but these are typically restricted to dose. Other formulations include empirically derived approximations of other radio-biologically relevant parameters, such as range or Linear Energy Transfer (LET) for a specific particle radiation type^19–21^. In this work, we take a different approach, starting from fundamental physics in the form of the Bethe equation to yield an analytical general solution for velocity, range, energy, and LET. This is broadly applicable for most charged particles used in therapy, with the notable exception of electrons, which violate important assumptions in the Bethe equation and are not considered in this work. The formulation here is suitable for heavier charged particles across the range of therapeutic energies. We compare results from this work with tabulated data, and MC simulations for protons, to ascertain how effective such an approach is at capturing the gross behaviour of such systems, and demonstrate potential application of the method outlined.

## Theoretical background

Charged particles undergo electronic interactions as they traverse a medium, losing energy in the process and slowing down. As the particle slows down, the density of ionizations induced in the medium increases, before. Ionization density drops abruptly to zero beyond the Bragg peak, since all of the particle’s kinetic energy has been exhausted and it can be considered stationary. Under certain assumptions, the stopping power in a medium is given by the relativistic Bethe equation^22^. Further corrections can be made to account for high energy particles and high atomic number targets, including the Barkas, Bloch and Fermi corrections, comprehensively reviewed by Ziegler^23^. However, at energies and in materials relevant to radiotherapy, these effects are expected to introduce errors less than 1% (less than 1 mm) whereas in radiotherapy, uncertainties greatly exceed this level^13^. Accordingly we neglect these corrections in order to achieve an analytical solution, without loss of accuracy in the regime of interest. The relevant form of the Bethe equation is then

1 $$-\frac{dE}{dx}=\frac{4\pi n{z}^{2}}{{m}_{e}{c}^{2}{\beta }^{2}}{(\frac{{e}^{2}}{4\pi {\varepsilon }_{o}})}^{2}(ln(\frac{2{m}_{e}{c}^{2}{\beta }^{2}}{I\mathrm{(1}-{\beta }^{2})})-{\beta }^{2})$$

where *n* is the electron density of the material, *e* the electron charge, *m*_*e*_ the electron mass, *I* the mean excitation potential, *z* the multiple of electron charge and $\beta =\frac{v}{c}$, where *v* is the speed of the particle and *c* is the speed of light in a vacuum. Particle energy is a function of particle speed, itself a function of distance. The quantity given by equation 1 can be considered the linear electronic stopping power of a medium for a given particle type, and represents the effect of the medium on the particle. The reciprocal effect of the particle on the medium is termed unrestricted linear energy transfer (LET); the two quantities are equal by the definitions in ICRU Report 85^24^. In general LET only considers collisions where the kinetic energy imparted to secondary electrons is below a given threshold (or restriction). This restricts the quantity to shorter-range electrons, which might provide better characterisation of radiation effects on the cellular scale. However, this study intends to characterise radiation on a millimetre scale, and microscopic dosimetry and radiobiological effects are considered outside its scope. It therefore follows many other medical physics investigations^25–27^ in considering unrestricted LET a relevant quantity for clinical modelling work. This is justifiable since calculations suggest a monotonic relationship between restricted and unrestricted LET^28^, and a number of radiobiological models^29, 30^ make use of unrestricted LET.

The range of a charged particle in a medium may be defined in a number of different ways, depending on the theoretical or experimental context. In this work, we focus on the continuous-slowing-down approximation (CSDA) range, obtained by integrating the inverse of Equation 1 between a particle’s initial energy and its stationary state. It should be noted that in practice the projected range (ie. the distance travelled by the particle along its initial direction) is slightly reduced by small deflections caused by scattering. However, the impact of this is minimal at clinical energies in low *Z* materials, with the ‘detour factor’ of the projected range compared to the CSDA range being greater than 99.87% for protons of energy >100 MeV in water^31^. Our definition of range is therefore, expressed in both energy and velocity terms as

2 $$R={\int }_{0}^{{E}_{0}}\frac{dx}{dE}dE={\int }_{0}^{{v}_{0}}\frac{dx}{dv}dv,$$

where the subscript 0 denotes the initial value at *x* = 0.

## Model derivation

We begin from the relativistic definition of particle kinetic energy, expressed in terms of the Lorentz factor:

3 $$E=(\gamma -\mathrm{1)}{m}_{p}{c}^{2},\quad \gamma ={(1-{\beta }^{2})}^{-\mathrm{1/2}},\quad \beta =\frac{v}{c}$$

From this, $\frac{dE}{dv}$ can be readily calculated, and using the chain rule $(\frac{dE}{dx}=\frac{dE}{dv}\frac{dv}{dx})$ it can be shown that

4 $$\frac{dE}{dx}={m}_{p}v(x){\gamma }^{3}\frac{dv}{dx}.$$

If equation 4 and equation 1 are equated directly, a solution for *v*(*x*) is analytically intractable. To circumvent this, we introduce a co-ordinate system *x*′ where the classical expression for particle kinetic energy of $E=\frac{{m}_{p}v{(x^{\prime} )}^{2}}{2}$ holds. Thus, we can write

5 $$\frac{dE}{dx^{\prime} }={m}_{p}v(x^{\prime} )\frac{dv}{dx^{\prime} }.$$

It is possible to simplify equation 1 by reducing the *β* ^2^ term, yielding

6 $$-\frac{dE}{dx}=\frac{4\pi n{z}^{2}}{{m}_{e}}{(\frac{{e}^{2}}{4\pi {\varepsilon }_{o}})}^{2}(\frac{1}{{v}^{2}})ln(\frac{2{m}_{e}{v}^{2}}{I}).$$

This is justified if $ln(\frac{2{m}_{e}{v}^{2}}{I\mathrm{(1}-{\beta }^{2})})-{\beta }^{2}\approx ln(\frac{2{m}_{e}{v}^{2}}{I})$. For a high energy proton at 250 MeV, there is only ≈1% difference between both sides, and ≈2% for high energy (400 MeV/a.m.u) carbon ions. This minor difference rapidly dissipates as the particles decelerate, and so the simplification can be employed with only minor discrepancy for particles in the radio-therapeutic energy range. For brevity, we let $A=\frac{4\pi n{z}^{2}}{{m}_{e}{m}_{p}}{(\frac{{e}^{2}}{4\pi {\varepsilon }_{o}})}^{2}$ and $B=\frac{2{m}_{e}}{I}$. We now define the CSDA range in the *x*′ coordinate system using an expression of similar form to Equation 2.

7 $$R^{\prime} ={\int }_{0}^{{v}_{0}}\frac{dx^{\prime} }{dv}dv,$$

Substituting equation 6 into equation 5, and the result thereof into equation 7, we get an expression similar to that reported by Evans^32^:

8 $$R^{\prime} ={\int }_{0}^{{v}_{0}}\frac{{v}^{3}}{A\,\mathrm{ln}(B{v}^{2})}dv.$$

This can be solved, and yields a solution in terms of the exponential integral function *Ei*. From this, it can thus be shown that particle range in the *x*′ coordinate system is given by

9 $$R^{\prime} =\frac{Ei(ln({B}^{2}{v}_{0}^{4}))}{2{B}^{2}A}.$$

If we change the lower limits on equation 7, we can readily show that $x^{\prime} =R^{\prime} -\frac{Ei(ln({B}^{2}v{(x^{\prime} )}^{4})}{2{B}^{2}A}$, and thus the solution for velocity *v*(*x*′) in terms of *x*′ is simply

10 $$v(x^{\prime} )=\frac{1}{\sqrt{B}}{\rm{e}}{\rm{x}}{\rm{p}}(\frac{1}{4}(E{i}^{-1}\mathrm{(2}{B}^{2}A(R^{\prime} -x^{\prime} )))).$$

The velocity formula in equation 10 is sufficient when $\gamma \simeq 1$, but in practice high-energy charged particles used in treatment often are far beyond this, and a better expression is required to account for them. To allow for relativistic effects, we can introduce a transform from *x*′ to *x*. We can re-arrange equations 4 and 5 and equate them; it can thereby be shown that *dx* = *γ* ^3^ *dx*′ and consequently that

11 $$x(x^{\prime} )={\int }_{0}^{x^{\prime} }{(1-{(\frac{v(x^{\prime} )}{c})}^{2})}^{-\mathrm{3/2}}dx^{\prime} .$$

This transform can be easily evaluated provided once *v*(*x*′) is known, and the transform yields the true solution *v*(*x*). In the *x* frame, the true CSDA range, *R*, is then given by

12 $$R={\int }_{0}^{R^{\prime} }{(1-{(\frac{v(x^{\prime} )}{c})}^{2})}^{-\mathrm{3/2}}dx^{\prime} .$$

**Figure 1:**
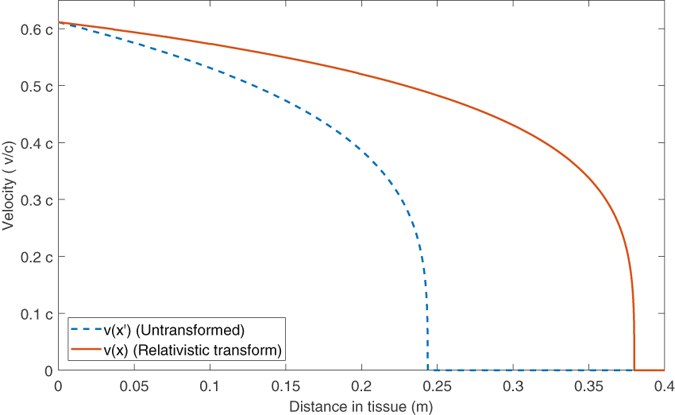
Comparison of *v*(*x*′) and *v*(*x*) (note Velocity shown on the vertical axis as a fraction of the speed of light *c*) for a 250 MeV proton. In this example, the projected range of the proton is 38.15 cm when the relativistic transform is considered. If this were neglected, projected range would be only 24.36 cm. This substantial difference suggests that taking account of the relativistic transform is vital for particles in the radiotherapy energy range.

This can also be calculated using a series expansion method, outlined in the appendix (Supplementary data). An illustration of the relativistic transform is depicted in Fig. 1. Once the transformed velocity profile is known, it can readily be applied to yield the mono-energetic energy curve and LET through identities 3 and 6 respectively.

### The inverse exponential integral function - *Ei*^−1^

The solution derived above requires knowledge of the inverse exponential integral function. This is not a standard function, and to the authors’ knowledge no closed form expression exists for it. Values can be readily derived using simple root-finding methods in most computational software packages. It is also possible to employ some simple approximations - for most instances the approximation *Ei* ^−1^(*x*) ≈ ln(*x*ln(*x*ln*x*)) can be used without introducing serious error, and Chebyshev polynomials can be employed to higher precision^33^, though knowledge of the limits of validity are important. A series expansion approach is also possible. For this work, we wished to plot the solution at high resolution (steps of 1 *μm*), and to speed this up we created a high precision look-up table using root-finding methods. The look up table, as well as examples of how to implement the model, are available in the Supplementary data.

## Methods

### Comparison with numerical methods

To ascertain how well the solution proposed fits the both forms of the Bethe equation, numerical solutions for equation 1 (fully relativistic) and 6 (simplified form) were found using a Runge-Kutta method. These solutions were contrasted with the analytical method outlined in this work, and the level of agreement between the two approaches was quantified. Physical parameters used in this work are given in Table 1.

**Table 1:** Parameters used for all simulations.

Parameter	Value (Proton)	Value (Carbon Ion)
Electron density *n*	3.343 × 10^29^ m^−3^	3.343 × 10^29^ m^−3^
Electron mass *m*_*e*_	1.672 × 10^−27^ kg	1.672 × 10^−27^ kg
Electron charge *e*	1.602 × 10^−19^ C	1.602 × 10^−19^ C
Vacuum permittivity *ε*_*o*_	8.854 × 10^−12^ F	8.854 × 10^−12^ F
Ionization Potential *I*	75 eV	75 eV
Atomic number *z*	1	6
Particle mass *m*_*p*_	1.673 × 10^−27^ kg	2.007 × 10^−26^ kg

### Comparison with tabulated data, Monte Carlo methods and experimental data

MC particle transport codes are traditionally used as the primary method for proton dose-depth calculations. We used the MCNPX v2.7.0 package^15^ to simulate proton pencil beams with a range of initial energies in water-like tissue and contrasted the predictions with the model outlined here. There has been less work done on carbon ions as these are less common in clinical use, though MC techniques using e.g. GEANT4 are emerging to explore this promising modality^34^. As implementing MC for carbon ions is still an area of active research, we have instead taken the measured range of carbon ions in water from various experimental investigations and contrasted this with the estimated range using the model to gauge the validity of model predictions for heavy ions like carbon.

The proton MC geometry comprised an infinitely narrow pencil beam of monoenergetic particles, incident on a water phantom with length 20% greater than the proton range. Phantoms of two different lateral cross sections were considered: a ‘broad’ 10 cm × 10 cm case, in which protons undergoing multiple Coulomb scattering (MCS) would be expected to stop, and a ‘narrow’ 1 mm × 1 mm case, in which the majority of protons undergoing MCS would be expected to escape. Mean energy and LET profiles were recorded at 1 mm intervals using surface tallies (MCNPX type F2, binned into 1000 subdivisions using the E card, modified to tally unrestricted stopping power by including the LET flag on an FT card). Only protons were tracked, from the source energy down to 1 keV. Default physics options were employed, with the addition of light-ion recoil. In addition to this, range estimates were compared to fitted data from the National Institute of Standards and Technology (NIST) PSTAR database for protons in liquid water^35^ and compared to results derived from this method. Carbon ion ranges were compared to tabulated results from ICRU report 73^36^ and the MSTAR^37^ software empirical estimation.

### Model applications

The chief advantage of charged particle therapy is that dose can be accurately delivered to a tumour region whilst largely sparing healthy tissue downstream, in contrast to photons which deposit dose relatively uniformly throughout the beam path. However, this advantage also presents a significant difficultly - dose must be well-aimed and delivered to very high precision^38^. If this is not the case, then the bulk of dose deposition could occur away from the tumour and into healthy organs, negating any benefit from therapy. To complicate matters, for example there is a six-fold difference between the electron density of inflated lung and cortical bone^39^ – further examples are given in Table 2. Contemporary fast dose calculation methods employ pencil beam algorithms, which account for heterogeneity by scaling dose-depth curves measured in water; such an approach does not provide estimates of LET, which is an important consideration for the radiobiological evaluation of treatment approaches^40^. The model here could be useful for such applications, as it can be applied to quickly estimate energy distribution through inhomogeneous media, such as tissue and we demonstrate this here by applying the model to inhomogeneous tissue derived from a typical CT scan as proof of principle.

**Table 2:** Electron densities of various tissue types (From Schneider *et al*.^39^).

Tissue	Electron density relative to water - *ρe*
Water	1.000
Muscle	1.040
Adipose tissue	0.957
Bone (Femur)	1.278
Bone (Rib)	1.347

Additionally, the model here can be used to explore the effect of the physical parameters on energy deposition profile. Most of the parameters required in this model are accurately known physical constants, as can be seen in Table 1. Of all these parameters however, there has been considerable variation in literature for the mean ionization potential for water. The value used of 75 eV is in line with the International Commission on Radiation Units and Measurement (ICRU) guidelines^36^, with a more recent ICRU report suggesting a lower value of 67 eV^41^. Other authors suggesting a much higher value of 80.2 ± 2 eV^42^ based on experimental range measurements. Using the model here, we can readily vary this and see what impact this has on range and energy profiles obtained.

## Results

### Comparison with numerical methods

**Figure 2:**
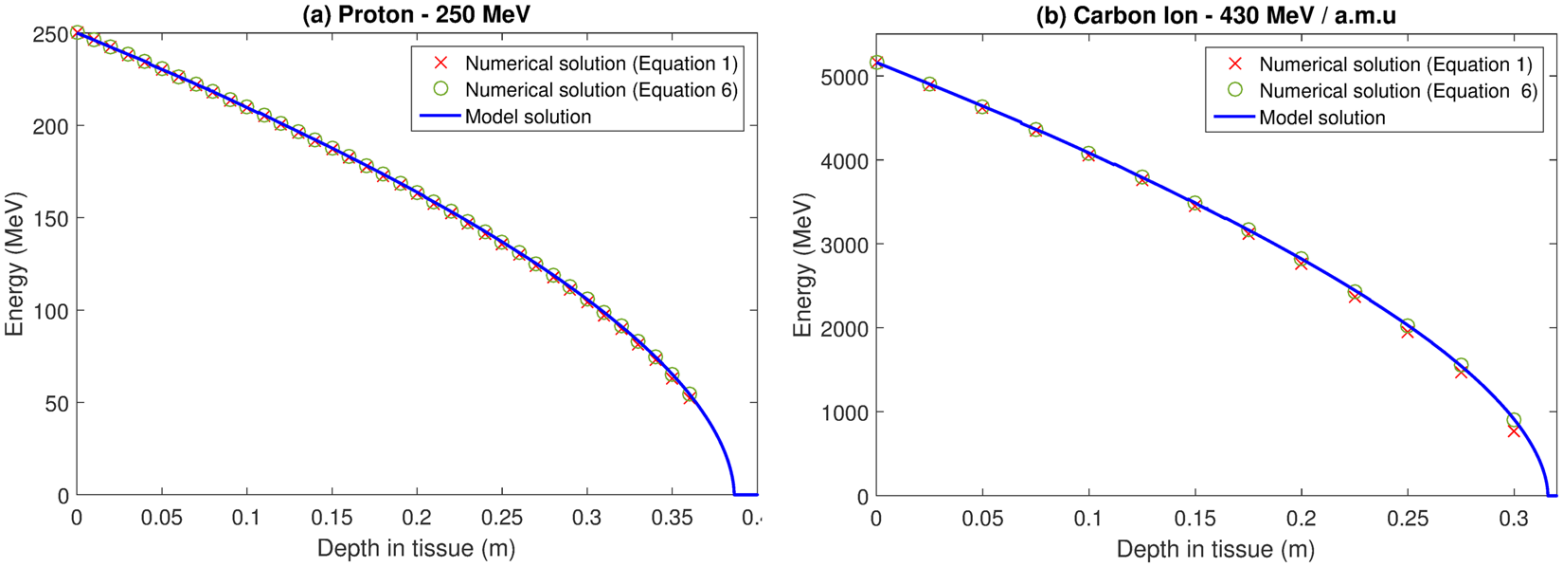
Comparison of model with numerical solution of the full Bethe equation (Equation 1) and the simplified form (Equation 6) for (**a**) a high energy proton and (**b**) a high energy Carbon ion. High energies are shown here as this is where maximum disagreement should manifest. As can be seen from the figure, the model matches the numerical solutions to a very high degree of accuracy, even at these high energies.

Equations 1 and 6 were solved using MATLAB ODE45, an adaptive step Runge-Kutta method. For protons the difference between the model and equation 6 was negligible, with a mean error of ≪1%. Even if the fully relativistic version of the Bethe equation is used (equation 1) errors tend to be minor; in the case of protons, the mean error between model and relativistic solution ranges from 0.049% at 100 MeV to 0.7800% at 250 MeV. For carbon ions, the mean error between the model and relativistic numerical solution ranges from 0.43% at 250 MeV/a.m.u to a high of 2.1584% at 430 MeV/a.m.u. This suggests the model accurately captures the dynamics of equation 6 and can describe the fully relativistic case in equation 1 with only minor error. Figure 2 depicts the solutions as a function of depth for protons and carbon ions at high initial energy, where discrepancy should manifest. Even here model predictions agree closely with numerical solutions, with no discernable difference between model predictions and numerical solutions of equation 6, and only minor deviation from equation 1 and the model over a realistic range of energies. This suggests that the model robustly captures the dynamics of the Bethe equation without recourse to numerical ODE solvers.

### Comparison with tabulated data, Monte Carlo methods and experimental data

**Figure 3:**
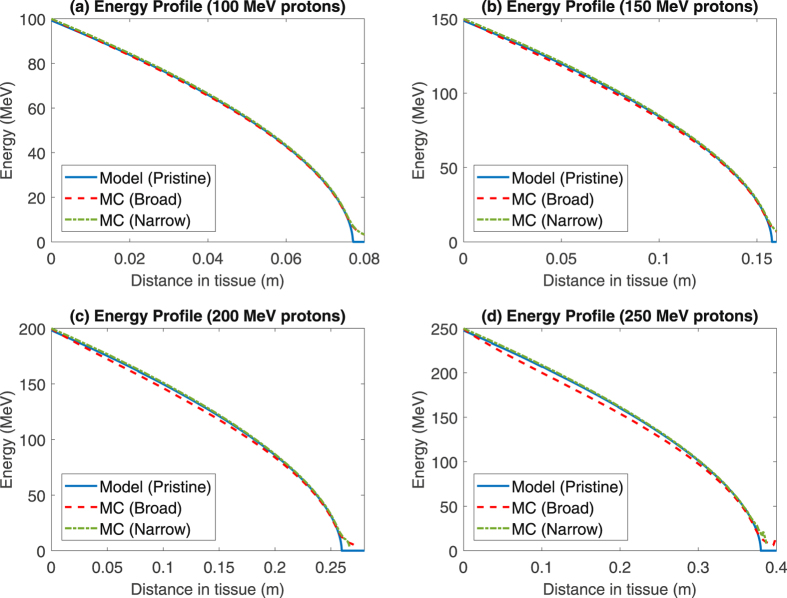
Comparison of pristine (mono-energetic) model predictions and MC simulations for proton mean energy as a function of depth, for protons with initial energies of 100–250 MeV in both broad and narrow cross section phantoms.

Figure 2 depicts the simulated energy profiles from the model established in this work and from traditional MC methods. In general there is good agreement between both approaches, quantified by the data in Table 3. The coefficient of determination column, *R* ^2^ compares the fit of the MC data with the model data, and predicted ranges, *R*_*T*_, are shown also. For the model, this was theoretically predicted by equation 12, and for MC simulations this was the depth beyond which no protons penetrated in the ‘broad’ phantom. While the ranges are in close agreement, there are some interesting differences. Most noticeable is that the MC simulation predicts a slightly lower energy along the curve, as quantified in the table. Secondly, it predicts a small tail that extends beyond the predicted *R*_*T*_ of the model. This is considered in the discussion, but most likely arises from the fact that a solution of the Bethe equation doesn’t consider energy and path length straggling, arising from stochastic effects and MCS respectively, or secondary charge particle production - all of which are incorporated in MCNPX. Further evidence for this is seen in comparing the curves in Fig. 3 for the ‘broad’ MC phantom to those for the ‘narrow’ phantom, where scatter effects are lower and the discrepancies between the model and simulation lesser. Table 3 also shows the percentage error between range estimated by method in this work and PSTAR NIST^35^ data, with errors of ≪1% between database and model.

**Table 3:** Model results versus PSTAR values and Monte Carlo simulations for protons.

Energy	*R*_*T*_ (Model)	*R*_*T*_ (PSTAR)	*R*_*T*_ (MC)	PSTAR Error	*R* ^2^	Mean error (MC)
100 MeV	7.72 cm	7.718 cm	8.1 cm	0.03%	0.9989	0.81 MeV
150 MeV	15.80 cm	15.77 cm	16.6 cm	0.19%	0.9977	1.96 MeV
200 MeV	26.05 cm	25.86 cm	27.2 cm	0.35%	0.9962	3.47 MeV
250 MeV	38.15 cm	37.94 cm	40.0 cm	0.55%	0.9931	5.89 MeV

**Figure 4:**
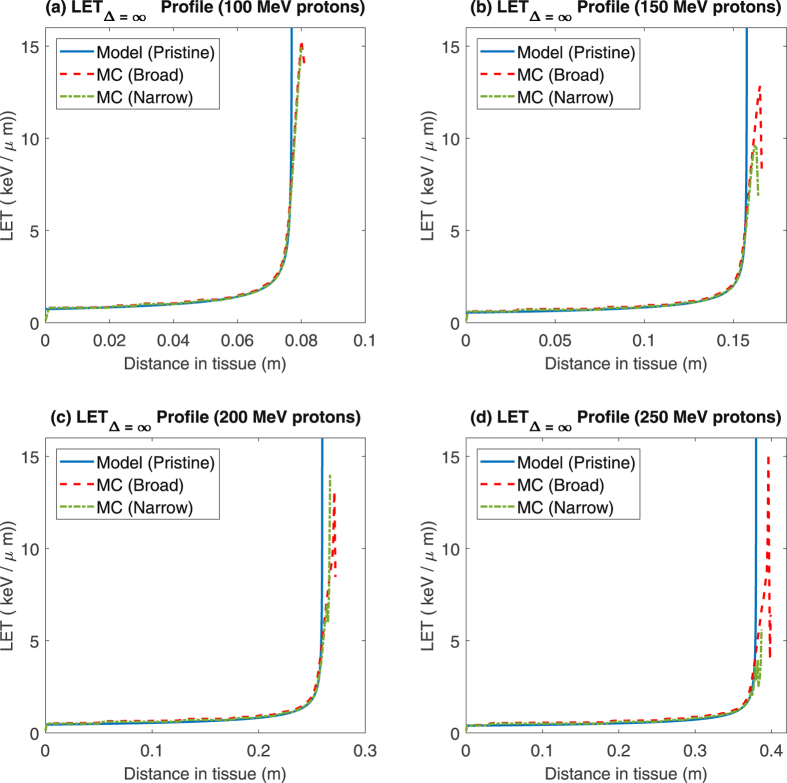
Comparison of pristine (mono-energetic) model predictions and and MC simulations for proton unrestricted LET as a function of depth, for protons with with initial energies of 100–250 MeV in both broad and narrow cross section phantoms.

The tail discrepancy is also obvious in the LET curves, depicted in Fig. 4. Obeying the Bethe equation, the model predicts a smooth asymptotic curve by definition from equations 1 and 3. The model closely matches the MC LET for most of the particle range, but diverges close to the terminal end of the track, as can be seen in the figure. This difference may be explained by straggling and secondary interactions, which the Bethe equation does not consider *a priori*.

**Figure 5:**
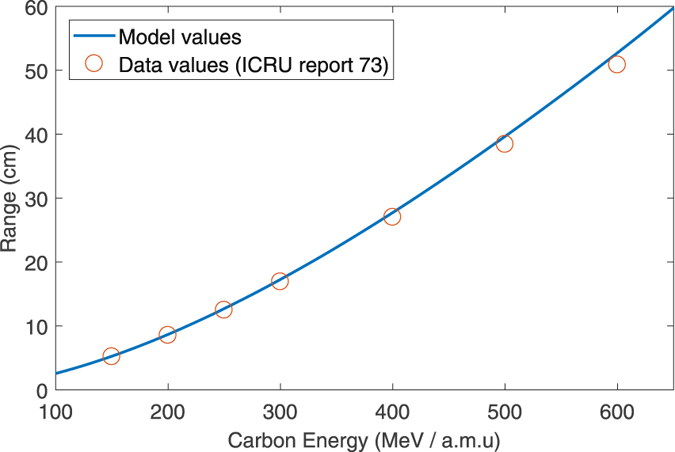
Comparison of tabulated range data for Carbon-ions from ICRU report 73 versus model predictions. High agreement was found along the full energy range, with *R* ^2^ = 0.9968 between model and data.

Table 4 shows tabulated and experimentally determined ranges for carbon ions using different methodologies. For ease of comparison, the experimental range was determined as the depth of the zenith of the Bragg peak. The predicted range from the model agrees within ≈1% of empirical values derived from MSTAR^37^, and also well with the experimentally determined values across all energy ranges between 135 MeV/a.m.u to 380.45 MeV/a.m.u with relative errors ranging from 0.21 % to 6.14 %. Part of this discrepancy might be apparent in 2(b), in which a small reduction in particle energy at a given depth is observed when using the full Bethe equation, compared to the simplified form. A further part is likely attributed to straggling and secondary interactions. Figure 5 depicts the model prediction for range versus the tabulated values in ICRU 73^36^, showing high agreement for carbon-ions in the radiotherapy range of 100–600 MeV/a.m.u, with a co-efficient of determination between model and tabulated data of 0.9968.

**Table 4:** Model results versus MSTAR and experimental results for carbon ions.

Energy	*RT* (Model)	*RT* (MSTAR)	*RT* (Experiment)	MSTAR Error (%)	Experiment Error (%)
135.00 MeV/u	4.38 cm	4.37 cm	4.43 cm^52^	0.23%	1.37%
195.00 MeV/u	8.31 cm	8.28 cm	8.34 cm^52^	0.36%	0.36%
208.58 MeV/u	9.33 cm	9.29 cm	8.79 cm^34^	0.43%	6.14%
241.50 MeV/u	11.99 cm	11.92 cm	11.87 cm^53^	0.58%	1.01%
270.00 MeV/u	14.48 cm	14.37 cm	14.45 cm^52^	0.76%	0.21%
279.97 MeV/u	15.39 cm	15.27 cm	14.73 cm^34^	0.78%	4.48%
330.00 MeV/u	20.24 cm	20.04 cm	19.98 cm^52^	0.99%	1.30%
332.15 MeV/u	20.46 cm	20.25 cm	19.74 cm^34^	1.03%	3.64%
380.45 MeV/u	25.57 cm	25.25 cm	24.76 cm^34^	1.25%	3.27%

### Model application

**Figure 6:**
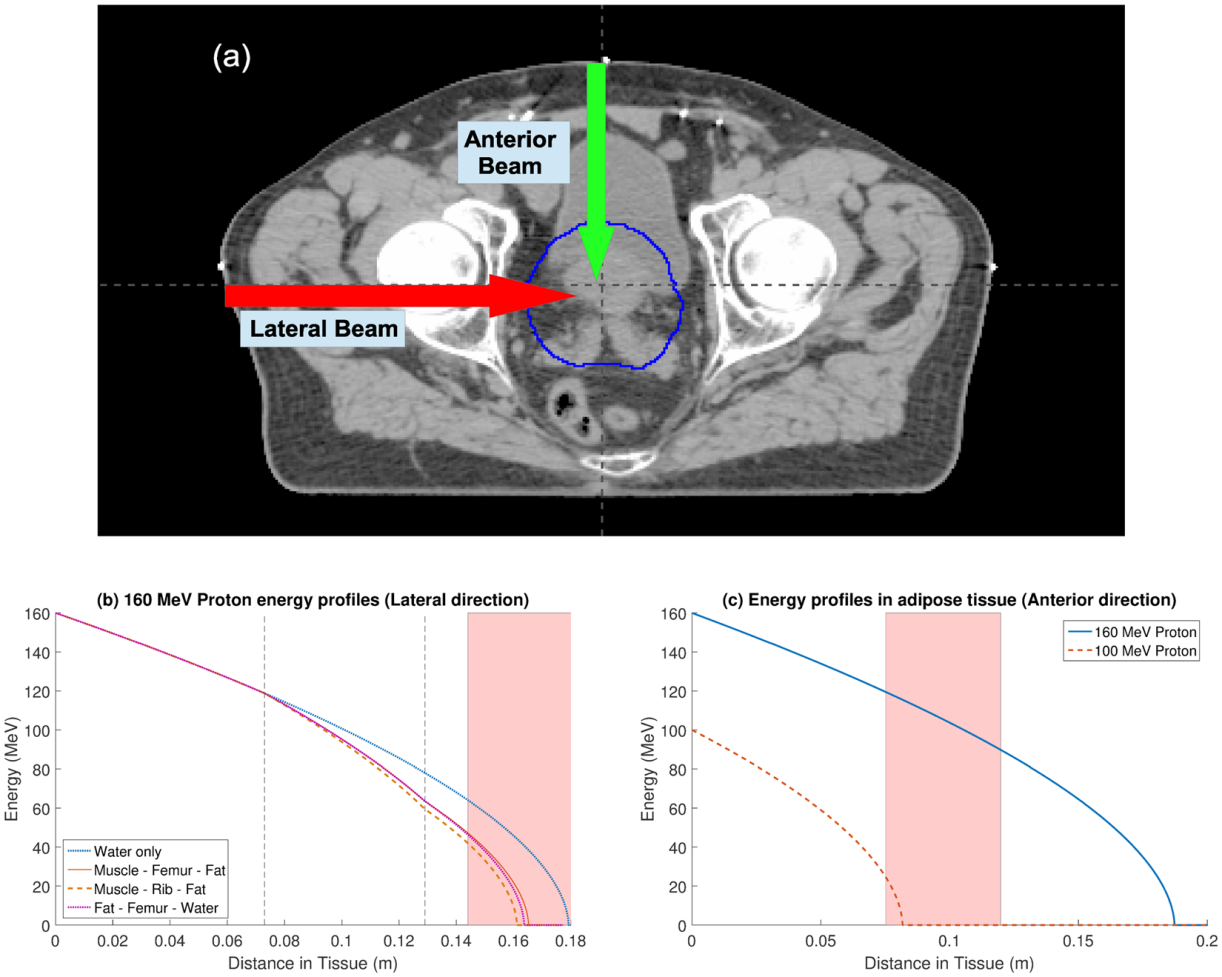
(**a**) Typical radiotherapy treatment planning scan for prostate cancer, with tumour volume outlined in blue. Possible anterior and lateral beam trajectories are indicated with arrows. A lateral beam passes through 7.3 cm of tissue before encountering 5.6 cm of bone and finally the organ. The anterior beam passes through a more uniform medium. (**b**) Energy profiles for 160 MeV protons, assuming different constituent tissues along the lateral beam trajectory. Regions of tumour are denoted by the shaded area and tissue interfaces by the vertical dashed lines (**c**) Energy profiles for protons entering along the anterior beam trajectory, with tumour region shaded. In this case, a 160 MeV proton would deposit the bulk of its energy downstream of the tumour into radio-sensitive tissue. By contrast, lower energy protons such as the 100 MeV path shown here would be sufficient to target the tumour volume.

A major motivation for the model outlined here is to rapidly simulate likely energy profiles for dose optimization. This model also readily allows for fast simulation of dose through different tissue types. A typical prostate radiotherapy planning scan is depicted in Fig. 6(a), with the planning target volume outlined in blue. This image slice shows at least three distinct regions, and the energy deposition was calculated with this model for hypothetical proton beams assuming varying tissue electron density as given in Table 2. The results of this are shown in Fig. 6(b) and (c) for proton and carbon ion plans respectively.

**Figure 7:**
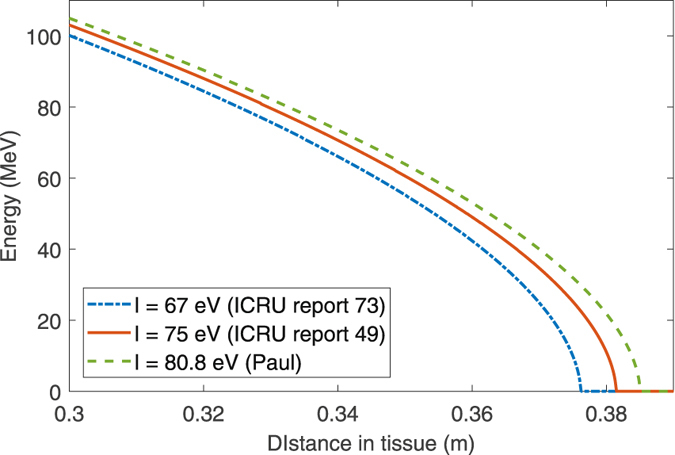
Energy profile for a 250 MeV proton in water with different choices of mean ionization potential, *I*, shown between 0.3 ≤ *x* ≤ *R*_*T*_. Profiles are initially similar, with divergence manifesting towards path end.

The energy profile for both proton and carbon ions with variation of mean ionization potential of water is shown in Fig. 7 for a 250 MeV proton. The standard ICRU value of 75 eV was used for simulations in this work, but Paul^42^ has suggested 80.8 eV better fits some experimental data. The net effect of an increasing estimate of *I* is an increase in observed range, and converse a decrease in range if lower estimates of *I* are employed. The physical explanation for this is relatively intuitive, as higher estimates for mean ionization potential translates to fewer ionization events and thus an increase in possible range.

## Discussion

The model presented here is an accurate and analytical solution to the Bethe equation - Fig. 1 shows how the continuous solution compares to that derived from conventional Runge-Kutta methods. Results from both are virtually identical, suggesting that the model captures the equation dynamics well. A further advantage of the analytical solution is that it explicitly yields a particle range *R*_*T*_ and consequently the Bethe equation-predicted energy, velocity and LET at any point throughout the track. When contrasted to MC simulations, Figs 2 and 3 show that the model captures the gross behaviour of a proton beam well but there are some important caveats to this. Primarily, the model slightly over-predicts particle energy along the track on average, with a mean error ranging from 0.81 MeV for 100 MeV protons up to 5.89 MeV for 250 MeV protons (Table 3), and discrepancies growing at an accelerating rate with particle energy. The most likely explanation for this is due to an inherent limitation of the Bethe equation, which does not intrinsically consider MCS or secondary (or higher order) production, which the MC simulation does. In particular, the magnitude of straggling has been observed to be approximately proportional to total particle range^4^ - a trend also displayed in the mean errors reported in Table 3. The result would be additional observed energy loss at a given depth in MC, compared to the Bethe equation. This interpretation is supported by the inclusion of a “narrow” MC simulation also shown in Fig. 3 - particles in this simulation undergo less scattering and the results under this constraint lie closer to the continuous model solution. Model ranges were exceptionally close to tabulated PSTAR estimates, with errors of ≪1% for protons.

This has an interesting consequence around at the end of the particle track (typically defined in medical physics literature as the distal edge), at the particle’s maximum range. As can be seen from Fig. 4, the Bethe equation predicts a asymptotic increase in LET at *R*_*T*_. The MC simulation, by contrast, agrees closely with model prediction right up until the Bragg peak, where higher order effects manifest. An important consequence of this is that the model (and indeed, the Bethe equation itself) does not capture the dynamics of particles at the distal edge completely, instead only approximating the general behaviour around this point. For carbon ions, the predicted ranges and experimentally measured Bragged Peaks agreed closely (mean percentage errors 0.21%–6.14%) and again the bulk of this discrepancy might be explained by fundamental limitations of the Bethe equation around the distal edge. Model agreement with MSTAR data was high (≈1% error) even to energies beyond those employed in therapy (600 MeV/a.m.u).

It is crucial to note that the solution outlined here is not intended as a replacement to conventional MC methods, but as a useful means of rapid optimization. As the analytical form presented here can be rapidly implemented with minimal computational costs, it could readily be applied in optimization when multiple iterations of dose calculation are needed. Using the model here, a huge array or potential doses can be quickly simulated, and narrowed down to only the most promising, which then can be more robustly simulated with MC techniques as a final calculation, potentially saving considerable time and expense. The other major advantage of the expression presented here is that it allows rapid simulation of complicated cases involving heterogeneous tissue, based on well-defined material constants such as electron density and mean ionization potential instead of water-equivalent stopping power ratios (SPRs). An example is shown in Fig. 6. This is readily implemented and, as it is analytical, the approximate dynamics of particles in even complex media can be readily estimated. There is still debate over the ideal value for mean ionization potential for water, and the model was also applied to investigate this. Figure 7 suggests that this difference chiefly manifests at the distal edge.

**Figure 8:**
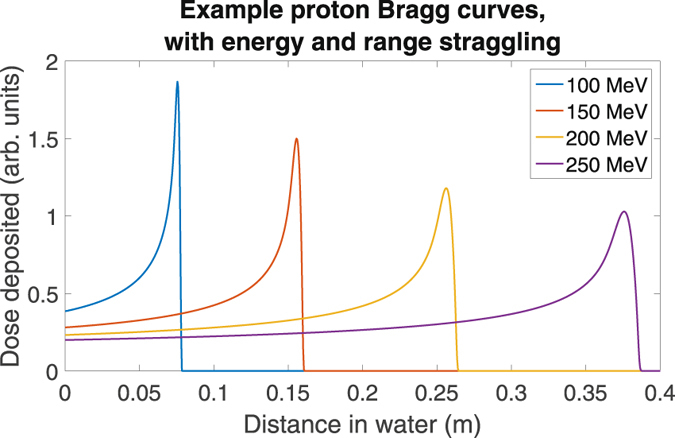
Example proton Bragg curves in water, found using calculations of proton kinetic energy as a function of path length from this work, combined with published formulations for energy and range straggling.

The method outlined here produces a solution for the Bethe equation, and hence the velocity, energy and LET curves for any charged particle rapidly from first principles. This is very useful, but it could potentially be extended to calculate Bragg curves in 1D by modelling energy variation and range straggling, or even in 3D by detailed consideration of MCS. These effects are the likely explanation for the departures between MC and model data seen at the distal edges in Figs 3 and 4. A number of semi-empirical formulations exist for multiple Coulomb scatter^43^ and energy variation^4^, but a detailed comparison of such approaches beyond the scope of this work. Examples of such Bragg curves are illustrated in Fig. 8, which were created using energy profiles from this model, a Gaussian approximation to the Vavilov energy straggling distribution^44^, and step-wise calculation of multiple Coulomb scatter using the Rossi formula^45, 46^. Additionally, the Bethe equation considers only stopping due to electrons within a material, but a full consideration of dose deposition mechanisms in tissue would also include nuclear effects.

For majority of the track length, the Monte Carlo LET agrees closely with the model as illustrated in Fig. 3. However, towards the distal edge the model (and Bethe equation itself) displays asymptotic behaviour. We can naively calculate the maximum obtainable LET from the Bethe equation presented in eq. 6 - defining this as *S*, we can readily calculate $\frac{dS}{dv}$. At some velocity *v*_*m*_ the particle is at its minimum speed and thus S tends to a maximum. Setting $\frac{dS}{dv}=0$, we can explicitly derive this velocity, given by

13 $${v}_{m}=\frac{{e}^{0.5}}{\sqrt{B}}=\sqrt{\frac{I{e}^{1}}{2{m}_{e}}}$$

This analysis yields *v*_*m*_ = 0.0141*c* for charged particles. The maximum possible LET for protons from the Bethe equation is thus 84.35 KeV/*μ*m and 3036.72 KeV/*μ*m for carbon in water. In reality, particles will not reach anything near this, due to deflections and energy losses from MCS, nuclear interactions and secondary production^4^. These are factors not intrinsically considered by Bethe equation or this model, and this in part explains the disparity between the model and Monte Carlo results at the distal edge as illustrated in Fig. 3. Tellingly, LETs are higher in the ‘narrow’ phantom Monte Carlo, reflecting that fact that maximum LET increases as the potential for scatter decreases.

It is important to note too that the Bethe equation is a continuous function, whereas charged particle interactions are in reality discrete events. Water and other discrete biological targets also have finite extent for energy transfer^47^, and we do not expect LET to reach these maximums. The resolution of the simulations is also worth considering; the model was evaluated continuously and plotted with a fine 1 micron resolution, whereas the MC has a much coarser scale of 1 mm. If a similar length scale is used for the model, the asymptotic behaviour at the distal edge disappears. The model presented is certainly not a substitute for MC calculations of LET, but captures the general behaviour well, though caution should be taken in interpreting results near the distal edge.

The ease and speed with which this formulation of the Bethe equation can be applied is a particular strength, as illustrated by the profiles shown in Fig. 6 for tissue of varying electromagnetic interaction properties. LET is also important in determining oxygen contribution to radiation damage, as molecular oxygen modulates the effectiveness of standard photon radiotherapy by up to a factor of three^48, 49^. It is known that oxygen enhancement varies markedly with LET^50^, and in principle accurate determination of LET for charged particles could be applied in conjunction with information on regions of chronic and acute hypoxia^51^, potentially improving treatment further. While this aspect remains speculative, the model presented in this work should lend itself to rapid approximation of particle range, energy and LET even in complex tissue, and ultimately should be of benefit in optimizing treatment and outcome.

## Conclusion

The solution presented here to the Bethe equation can be rapidly applied to any charged particle type, and yields accurate estimates of energy profile, velocity and LET through a medium. These are shown to agree well with experimental and Monte Carlo results for both protons and carbon ions, with some discrepancy at the distal edge. The approach outlined also lends itself to the rapid calculation of dose profile through different media, and could serve as a basis for rapid dose optimization for charged particle therapy.

## Electronic supplementary material

Supplementary information

## References

[1] Brown A, Suit H (2004). The centenary of the discovery of the Bragg peak. Radiotherapy and Oncology.

[2] Wilson RR (1946). Radiological use of fast protons. Radiology.

[3] Pedroni E (1995). The 200-MeV proton therapy project at the Paul Scherrer Institute: conceptual design and practical realization. Medical physics.

[4] Newhauser WD, Zhang R (2015). The physics of proton therapy. Physics in Medicine & Biology.

[5] Lomax AJ, Pedroni E, Rutz HP, Goitein G (2004). The clinical potential of intensity modulated proton therapy. Zeitschrift für Medizinische Physik.

[6] Schulz-Ertner D, Tsujii H (2007). Particle radiation therapy using proton and heavier ion beams. Journal of Clinical Oncology.

[7] Schlaff CD, Krauze A, Belard A, O’Connell JJ, Camphausen Ka (2014). Bringing the heavy: carbon ion therapy in the radiobiological and clinical context. Radiation oncology (London, England).

[8] Allen AM (2012). An evidence based review of proton beam therapy: The report of ASTRO’s emerging technology committee. Radiotherapy and Oncology.

[9] De Ruysscher D (2012). Charged particles in radiotherapy: A 5-year update of a systematic review. Radiotherapy and Oncology.

[10] Goitein M (2010). Trials and tribulations in charged particle radiotherapy. Radiotherapy and Oncology.

[11] Tsujii H, Kamada T (2012). A review of update clinical results of carbon ion radiotherapy. Japanese Journal of Clinical Oncology.

[12] Schulz RJ, Kagan AR (2016). Carbon-Ion Therapy: One More Step in the Endless Quest for the Ideal Dose Distribution. International Journal of Radiation Oncology Biology Physics.

[13] Paganetti H (2012). Range uncertainties in proton therapy and the role of Monte Carlo simulations. Phys Med Biol.

[14] Agostinelli S (2003). Geant4—a simulation toolkit. Nuclear Instruments and Methods in Physics Research Section A: Accelerators, Spectrometers, Detectors and Associated Equipment.

[15] Pelowitz, D. B. *et al*. MCNPX 2.7.0 Extensions. http://mcnpx.lanl.gov/opendocs/versions/v270/v270.pdf (2011).

[16] Ferrari, A., Sala, P. R., Fasso, A. & Ranft, J. FLUKA: A multi-particle transport code (Program version 2005) (2005).

[17] Bortfeld T, Schlegel W (1996). An analytical approximation of depth-dose distributions for therapeutic proton beams. Physics in medicine and biology.

[18] Bortfeld T (1997). An analytical approximation of the Bragg curve for therapeutic proton beams. Medical physics.

[19] Wilkens JJ, Oelfke U (2003). Analytical linear energy transfer calculations for proton therapy. Medical physics.

[20] Sanchez-Parcerisa D (2016). Analytical calculation of proton linear energy transfer in voxelized geometries including secondary protons. Physics in medicine and biology.

[21] Marsolat F, De Marzi L, Pouzoulet F, Mazal A (2016). Analytical linear energy transfer model including secondary particles: calculations along the central axis of the proton pencil beam. Physics in medicine and biology.

[22] Bethe H (1932). Bremsformel für Elektronen relativistischer Geschwindigkeit. Zeitschrift für Physik.

[23] Ziegler JF (1999). Stopping of energetic light ions in elemental matter. Journal of Applied Physics.

[24] Thomas DJ (2012). Icru report 85: fundamental quantities and units for ionizing radiation. Radiation Protection Dosimetry.

[25] Grassberger C, Paganetti H (2011). Elevated let components in clinical proton beams. Physics in Medicine and Biology.

[26] Bauer J (2014). Integration and evaluation of automated monte carlo simulations in the clinical practice of scanned proton and carbon ion beam therapy. Physics in Medicine and Biology.

[27] Guan, F. *et al*. Analysis of the track-and dose-averaged let and let spectra in proton therapy using the geant4 monte carlo code. *Medical physics* **42**, 6234–6247 (2015). 10.1118/1.4932217 PMC4600086 26520716

[28] Heinrich, W, Wiegel, B & Kraft, G. *β*, *Z*_*eff*_, dE/dx, range and restricted energy loss of heavy ions in the region 1 ≤ *E* ≤ 1000 MeV/Nucelon. GSI preprint Accessed 15th June 2017 (1991).

[29] Hawkins RB (1998). A microdosimetric-kinetic theory of the dependence of the rbe for cell death on let. Medical physics.

[30] Wilkens J, Oelfke U (2004). A phenomenological model for the relative biological effectiveness in therapeutic proton beams. Physics in medicine and biology.

[31] Paganetti H (1993). Relative biological effectiveness (RBE) values for proton beam therapy. Variations as a function of biological endpoint, dose, and linear energy transfer. Physics in medicine and biology.

[32] Evans, R. D. & Noyau, A. *The atomic nucleus*, vol. 582 (McGraw-Hill New York, 1955).

[33] Pecina P (1986). On the function inverse to the exponential integral function. Bulletin of the Astronomical Institutes of Czechoslovakia.

[34] Farina E (2015). Geant4 simulation for a study of a possible use of carbon ion pencil beams for the treatment of ocular melanomas with the active scanning system at CNAO. Journal of Physics: Conference Series.

[35] Berger, M., Coursey, J., Zucker, M. & Chang, J. Estar, pstar, and astar: Computer programs for calculating stopping-power and range tables for electrons, protons, and helium ions (version 1.2.3) (2005).

[36] Paul, H. *et al*. Stopping of ions heavier than helium (2005). 10.1093/jicru/ndi001 24170851

[37] Paul H, Schinner A (2001). An empirical approach to the stopping power of solids and gases for ions from 3 li to 18 ar. Nuclear Instruments and Methods in Physics Research Section B: Beam Interactions with Materials and Atoms.

[38] Paganetti H (2014). Relative biological effectiveness (RBE) values for proton beam therapy. Variations as a function of biological endpoint, dose, and linear energy transfer. Physics in medicine and biology.

[39] Schneider U, Pedroni E, Lomax A (1996). The calibration of CT Hounsfield units for radiotherapy treatment planning. Physics in medicine and biology.

[40] Underwood T (2016). Can we advance proton therapy for prostate? Considering alternative beam angles and RBE variations when comparing against IMRT. International Journal of Radiation Oncology*Biology*Physics.

[41] Powers, S. Ranges for protons and alpha particles. icru report 49. *Bethesda*, *MD* (2005).

[42] Paul H (2007). The mean ionization potential of water, and its connection to the range of energetic carbon ions in water. Nuclear Instruments and Methods in Physics Research, Section B: Beam Interactions with Materials and Atoms.

[43] Gottschalk B (2010). On the scattering power of radiotherapy protons. Medical Physics.

[44] Vavilov PV (1957). Ionization Losses of High-Energy Heavy Particles. Soviet Physics JETP.

[45] Soukup M, Fippel M, Alber M (2005). A pencil beam algorithm for intensity modulated proton therapy derived from Monte Carlo simulations. Physics in medicine and biology.

[46] Rossi B, Greisen K (1941). Cosmic-Ray Theory. Reviews of Modern Physics.

[47] Van den Heuvel F (2014). A closed parameterization of dna-damage by charged particles, as a function of energy - a geometrical approach. PLOS ONE.

[48] Grimes DR, Partridge M (2015). A mechanistic investigation of the oxygen fixation hypothesis and oxygen enhancement ratio. Biomedical Physics and Engineering Express.

[49] Grimes, D. R., Warren, D. R. & Warren, S. Hypoxia imaging and radiotherapy: bridging the resolution gap. *The British Journal of Radiology* **0**, 20160939 (0), doi:10.1259/bjr.20160939. PMID: 28540739. 10.1259/bjr.20160939 PMC5603947 28540739

[50] Wenzl T, Wilkens JJ (2011). Modelling of the oxygen enhancement ratio for ion beam radiation therapy. Physics in Medicine and Biology.

[51] Grimes, D. R. *et al*. Estimating oxygen distribution from vasculature in three-dimensional tumor tissue. *Journal of The Royal Society Interface* **13**, doi:10.1098/rsif.2016.0070 (2016). 10.1098/rsif.2016.0070 PMC4843681 26935806

[52] Sihver L, Schardt D, Kanai T (1998). Depth-Dose Distributions of High-Energy Carbon, Oxygen and Neon Beams in Water. Japanese Journal of Medical Physics.

[53] Yamamoto S, Komori M, Koyama S, Toshito T (2016). Luminescence imaging of water during alpha particle irradiation. Nuclear Instruments and Methods in Physics Research Section A: Accelerators, Spectrometers, Detectors and Associated Equipment.

