# Supplementary material for: An approximate analytical solution of the Bethe equation for charged particles in the radiotherapeutic energy range
Source: Sci Rep. 2017 Aug 29;7:9781. doi: 10.1038/s41598-017-10554-0 (PMC5574894; doi:10.1038/s41598-017-10554-0)
Supplement: Supplementary file 1 — Supplementary information [file 41598_2017_10554_MOESM1_ESM.pdf]

An approximate analytical solution of the Bethe equation for charged particles in the radiotherapeutic energy range

David Robert Grimes[1], Daniel R Warren[1], and Mike Partridge[1]

[1]Cancer Research UK/MRC Oxford Institute for Radiation Oncology, Gray Laboratory, University of Oxford, Old Road Campus Research Building, Off Roosevelt Drive, Oxford OX3 7DQ, UK
[*]davidrobert.grimes@oncology.ox.ac.uk

ABSTRACT

Charged particles such as protons and carbon ions are an increasingly important tool in radiotherapy. There are however unresolved physics issues impeding optimal implementation, including estimation of dose deposition in non-homogeneous tissue, an essential aspect of treatment optimization. Monte Carlo (MC) methods can be employed to estimate radiation profile, and whilst powerful, these are computationally expensive, limiting practicality. In this work, we start from fundamental physics in the form of the Bethe equation to yield a novel approximate analytical solution for particle range, energy and linear energy transfer (LET). The solution is given in terms of the exponential integral function with relativistic co-ordinate transform, allowing application at radiotherapeutic energy levels (50-350 MeV protons, 100 - 600 Mev / a.m.u carbon ions). Model results agreed closely for protons and carbon-ions (mean error within $\approx 1\%$) of literature values. Agreement was high along particle track, with some discrepancy manifesting at track-end. The model presented has applications within a charged particle radiotherapy optimization framework as a rapid method for dose and LET estimation, capable of accounting for heterogeneity in electron density and ionization potential.

Supplementary information

Ei^{-1} look-up table and code implementation

A finely resolved look-up table for the inverse exponential function and implementations of the model can be found at GitHub at https://github.com/drg85/CarbonProtonSolver. This code is in MATLAB format, and is easily portable to other languages and frameworks.

Series solution to simplified Bethe equation

It is possible to find a convergent series solution for the residual range of a particle following the simplified Bethe relation in equation 6, without requiring a transformation from the medium rest frame coordinate x. We start from the equivalent version of equation 8 in terms of x:

$$\int_0^{v_0} \frac{v^3}{\ln(Bv^2)} \mathrm{d}v = -\int_{R_T}^{0} \frac{A}{\gamma(v)^3} \mathrm{d}x \tag{1}$$

which may be arranged as

$$R_T = \frac{1}{A} \int_0^{v_0} \left[1 - \left(\frac{v}{c}\right)^2\right]^{-3/2} \cdot \frac{v^3}{\ln(Bv^2)} \mathrm{d}v \tag{2}$$

The series expansion of the square-bracketed term is:

$$\left[1 - \left(\frac{v}{c}\right)^2\right]^{-3/2} = \sum_{n=0}^{\infty} \frac{(2n+2)!}{2^{2n+1} \cdot (n+1)! \cdot n!} \left(\frac{v}{c}\right)^{2n} \tag{3}$$

The coefficient premultiplying $\left(\frac{v}{c}\right)^{2n}$ in equation 3 will hereafter be denoted F_n. Equation 2 can therefore be rewritten as a series of exponential integrals:

$$R_T = \frac{1}{A} \sum_{n=0}^{\infty} \frac{F_n}{c^{2n}} \int_0^{v_0} \frac{v^{3+2n}}{\ln(Bv^2)} \mathrm{d}v \tag{4}$$

or, in terms of the exponential integral function Ei:

$$R_T = \frac{1}{2A} \sum_{n=0}^{\infty} \frac{F_n}{B^{n+2} c^{2n}} \mathrm{Ei}\left[(n+2) \cdot \ln\left(B v_0^2\right)\right] \tag{5}$$

The first four values of F_n are 1, $\frac{3}{2}$, $\frac{15}{8}$, $\frac{35}{16}$ (for $n = 0$ to $n = 3$). The first term of equation 5 with $n = 0$ is therefore equivalent to the classical solution in main text equation 8. By manipulation of the limits in main text equation 12, it is additionally possible to show that the distance travelled by a particle in decelerating from v_0 to v is:

$$x(v) = R_T - \frac{1}{A^*} \sum_{n=0}^{\infty} \frac{F_n}{2B^{n+2} c^{2n}} \mathrm{Ei}\left[(n+2) \cdot \ln\left(B v^2\right)\right] \tag{6}$$

This is not readily invertible to obtain an explicit equation for $v(x)$, but it would be simple to approximate that function using a numerical look-up table.
